# CD8 T cell dynamics and immune cell trafficking in ZIKV infection: implications for neuroinflammation and therapy

**DOI:** 10.1186/s12985-025-02866-9

**Published:** 2025-07-15

**Authors:** Sungjun Park

**Affiliations:** Center for Infectious Disease Vaccine and Diagnosis Innovation (CEVI), Korea Research Institute of Chemical Technology (KRICT), Daejeon, Republic of Korea

**Keywords:** Zika virus, CD8 T cells, Flaviviruses, Immune cells infiltration, Central nervous system, Neuroinflammation, FTY720

## Abstract

The 2015–2016 Zika virus (ZIKV) epidemic underscored the severe consequences of congenital Zika syndrome (CZS) and the broader challenges posed by neurotropic flaviviruses. As key mediators of cytotoxic immunity, CD8 T cells play a crucial and multifaceted role in ZIKV pathogenesis. While essential for controlling viral replication, their infiltration into the central nervous system (CNS)—an immune-privileged site—raises potential concerns regarding immunopathology. This review explores the dual roles of CD8 T cells during ZIKV infection, emphasizing both their antiviral functions and their potential to drive neuroinflammation. We examine how ZIKV infection and chemokine-mediated signals facilitate immune cell trafficking across the blood–brain barrier, drawing parallels with other neurotropic flaviviruses. We also explore how therapeutic agents, such as the S1P receptor modulator FTY720, influence lymphocyte trafficking and CNS immune regulation. Finally, we review emerging interventions—including vaccines, antivirals, immunomodulators, and passive immunotherapies—that aim to achieve effective viral control while minimizing neural damage. A balanced understanding of immune cell responses in flavivirus infections is essential for guiding future therapeutic strategies against ZIKV and related neurotropic viruses.

## Introduction

Orthoflaviviruses (formerly known as Flaviviruses), a genus of positive-sense single-stranded RNA viruses, include several medically important human pathogens such as ZIKV, West Nile virus (WNV), Japanese encephalitis virus (JEV), and Dengue virus (DENV) [1]. Their ~ 11-kb genome contains a single open reading frame (ORF) flanked by 5′ and 3′ untranslated regions (UTRs), which encodes a polyprotein that is cleaved into three structural proteins (C: capsid, prM: precursor membrane, E: envelope) and seven nonstructural proteins (NS1–NS5) [2, 3]. The structural proteins mediate virion assembly and host cell entry, while the nonstructural proteins facilitate viral RNA replication and modulate host immune responses. NS3, with its cofactor NS2B, serves as a serine protease that processes the viral polyprotein. NS5 has two enzymatic domains: an N-terminal methyltransferase (MTase) for RNA capping, and a C-terminal RNA-dependent RNA polymerase (RdRp) for genome replication. Viral replication occurs in ER-derived membranous compartments that support efficient RNA synthesis and virion assembly [4, 5].

While these viruses primarily cause febrile illnesses and systemic symptoms, some flaviviruses—particularly ZIKV, WNV, and JEV—exhibit neurotropic potential, leading to severe neurological complications such as encephalitis, meningitis, Guillain-Barré syndrome, and congenital malformations [6–8]. Their ability to breach the blood–brain barrier (BBB) and infect CNS tissues [9–11] has prompted extensive investigation into the host immune responses that mediate both protection and pathology in the CNS.

Among immune effectors, CD8 cytotoxic T lymphocytes (CTLs) play a crucial role in controlling viral infections by eliminating infected cells and orchestrating antiviral responses through the secretion of cytokines such as interferon-γ (IFN-γ) and tumor necrosis factor-α (TNF-α) [12]. In peripheral tissues, CD8 T cell responses are often associated with viral clearance and protective immunity [13]. However, within immune-privileged sites like the CNS, their roles are far more complex. CD8 T cells can contribute to viral clearance but may also drive tissue damage and neuroinflammation due to the limited regenerative capacity of neurons and the tightly regulated nature of the CNS environment [14, 15].

In the context of neurotropic flavivirus infections, accumulating evidence suggests that CD8 T cells are recruited to the brain and spinal cord following viral invasion and may serve dual functions: promoting viral clearance while also contributing to neuropathology [14, 16–18]. Because the CNS has limited regenerative capacity, an imbalanced or excessive CD8 T cell response can aggravate neuroinflammation, promote neuronal damage, and ultimately contribute to persistent neurological sequelae [19–21].

Notably, the emergence of ZIKV as a global health threat in 2015–2016, particularly in Brazil, underscored the virus’s devastating neurotropic and teratogenic properties. The epidemic sparked widespread public fear due to its association with CZS, including microcephaly and other severe fetal brain malformations [22, 23]. This global crisis accelerated research into the immune mechanisms involved in ZIKV-induced neurological disease. In 2017, three independent research groups provided key insights into the role of CD8 T cells during ZIKV infection [24–26]. Their findings demonstrated that CD8 T cells play a protective role in antiviral defense and may contribute to the development of long-term immunity.

Understanding the precise contributions of antiviral versus pathogenic CD8 T cell activities is therefore critical, not only for the development of targeted therapies but also for designing vaccines that can elicit protective immunity without inducing harmful neuroimmune responses. This review synthesizes current knowledge on the roles of CD8 T cells during neurotropic flavivirus infections, with a particular emphasis on ZIKV. We highlight how CD8 T cells infiltrate the CNS, their phenotypic and functional characteristics during infection, and their dual potential to either protect against or exacerbate viral pathogenesis. Our goal is to provide a comprehensive framework for understanding how antiviral T cell responses are shaped within the brain and how they might be therapeutically harnessed or modulated to achieve optimal outcomes during flavivirus infections.

### CD8 T cells: central players in adaptive antiviral defense

CD8 T cells, also known as CTLs, are central effectors of the adaptive immune system, specialized in targeting and eliminating cells harboring intracellular pathogens, particularly viruses. Their activation is orchestrated by dendritic cells (DCs), which serve as key antigen-presenting cells in antiviral immunity. Upon capturing viral antigens, DCs process and present them on major histocompatibility complex (MHC) class I molecules, initiating the activation and clonal expansion of naïve CD8 T cells. This interaction drives their differentiation into effector CTLs capable of recognizing and killing virus-infected cells [27, 28]. In parallel, DC-derived cytokines play a crucial role in modulating the magnitude and quality of the CD8 T cell response by promoting their activation, survival, and functional maturation during the course of viral infection [29].

Effector CD8 T cells eliminate infected cells through two main mechanisms: direct cytotoxicity and the secretion of pro-inflammatory cytokines. Their cytotoxic activity is primarily mediated through the perforin–granzyme pathway, in which perforin forms pores in the target cell membrane, facilitating the entry of granzymes—particularly granzyme B—which then initiate caspase-dependent or -independent apoptosis [30–32]. A second major mechanism involves Fas ligand (FasL), expressed on T cells, binding to Fas receptors on target cells and triggering extrinsic apoptotic signaling via the caspase-8 pathway [33]. CD8 T cells can also engage the TRAIL (TNF-related apoptosis-inducing ligand)–DR5 (death receptor 5) pathway, contributing to apoptosis in infected or stressed target cells [34]. These multiple cytotoxic pathways can act synergistically or compensatorily depending on the viral context and the immune environment.

Beyond direct cytolysis, CD8 T cells secrete cytokines such as IFN-γ and TNF-α. These cytokines amplify the immune response by activating macrophages, enhancing antigen presentation, and inhibiting viral replication in both infected and bystander cells [35]. In addition to IFN-γ and TNF-α, CD8 T cells can produce other cytokines and chemokines that contribute to antiviral defense. Although IL-2 is predominantly produced by CD4 T cells [36], CD8 T cells can also intrinsically express IL-2. This intrinsic IL-2 production has been shown to promote CD8 T cell proliferation, support memory formation, and enhance antiviral protection by preventing exhaustion and sustaining long-term immunity [37]. Additionally, chemokines such as CCL3 (MIP-1α), CCL4 (MIP-1β), and CCL5 (RANTES) aid in the recruitment of other immune cells to the site of infection [38–40]. Moreover, CD8 T cell-derived granulocyte-macrophage colony-stimulating factor (GM-CSF) has been shown to enhance the function of antigen-presenting cells and bolster local immune responses against viral pathogens [41]. In certain viral infections, CD8 T cell-derived cytokines are essential not only for viral clearance but also for shaping the quality of the ensuing immune response [42].

Several studies using ZIKV infection models have shown that CD8 T cells play a protective role in antiviral defense. In particular, Elong Ngono et al. (2017) demonstrated that ZIKV-specific CD8 T cells are essential for controlling viral replication and mediating protection [24]. Similarly, Pardy et al. (2017) reported that CD8 T cells exhibit effector characteristics, including the secretion of IFN-γ and TNF-α, which may contribute to antiviral responses [26]. Moreover, the identification of ZIKV-specific epitopes recognized by CD8 T cells suggests that these cells not only mediate the destruction of infected targets but also contribute to the establishment of long-lasting immunity [24–26]. Indeed, the adoptive transfer of CD8 T cells from ZIKV-infected mice led to a reduction in viral burden, particularly in the brain [24, 25], indicating that CD8 T cells may also play a role in modulating ZIKV-associated neuropathology.

**Fig. 1:**
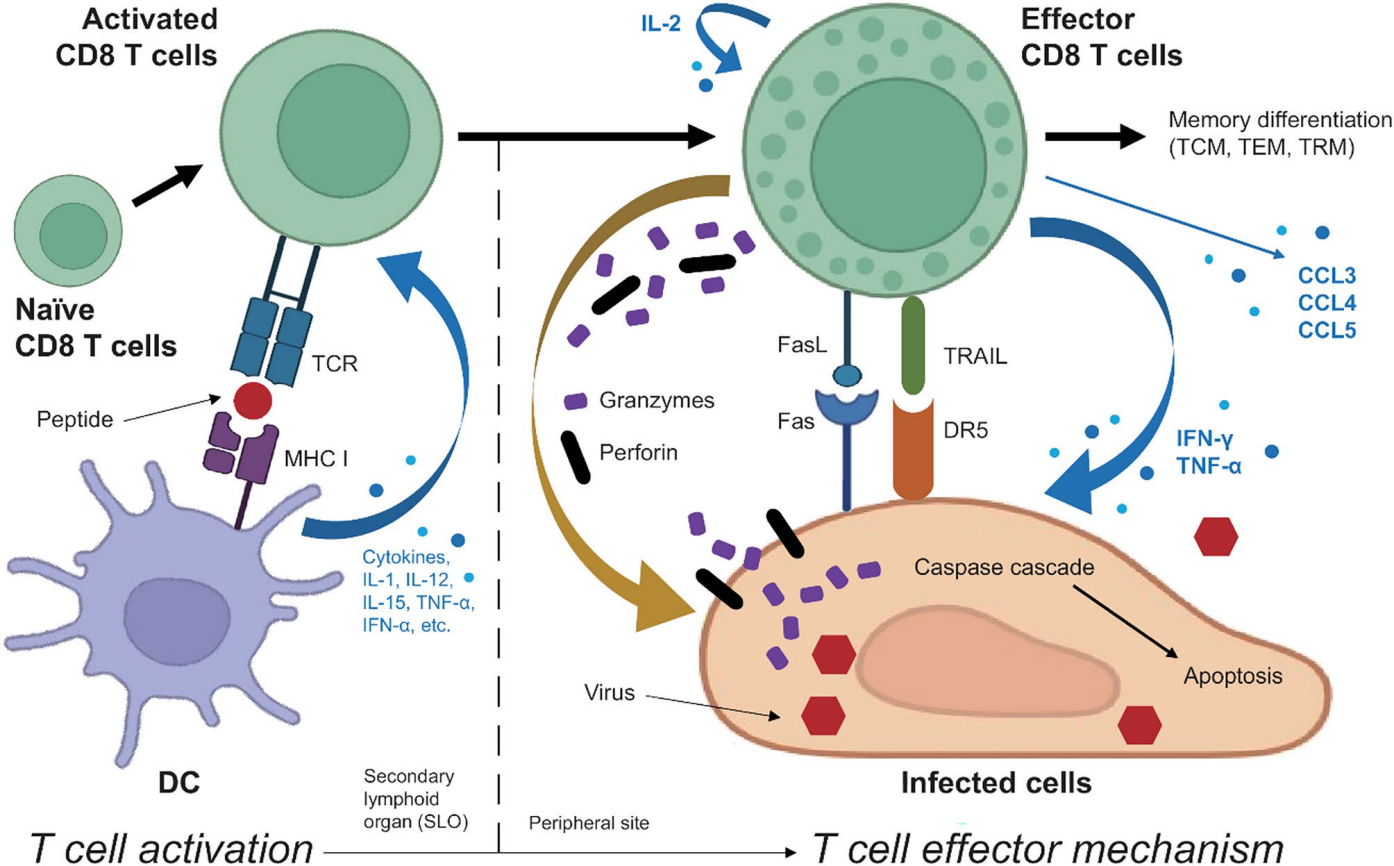
CD8 T cells are central effectors of antiviral adaptive immunity. CD8 T cells are key players in antiviral defense. Upon activation by viral peptides presented on MHC class I molecules, naïve CD8 T cells differentiate into effector cells that eliminate infected cells via perforin–granzyme, FasL–Fas, and TRAIL–DR5 apoptotic pathways. They also secrete cytokines (e.g., IFN-γ, TNF-α, IL-2) and chemokines (e.g., CCL3/4/5) that amplify immune responses and inhibit viral replication. A subset of these cells persists as memory CD8 T cells—including TCM, TEM, and TRM—providing long-term protection and rapid recall responses upon reinfection

Following antigen clearance and resolution of acute infection, the majority of activated effector CD8 T cells undergo programmed cell death during the contraction phase. However, a subset differentiates into long-lived memory CD8 T cells, which can be categorized into central memory (TCM), effector memory (TEM), and tissue-resident memory (TRM) subsets based on phenotype, function, and localization [43]. These memory populations provide long-term protective immunity and form the cellular basis of durable vaccine-induced protection [44]. Indeed, memory CD8 T cells are generated during ZIKV infection, and adoptive transfer studies have shown that these cells are sufficient to confer protection against lethal ZIKV challenge [24, 25]. Thus, the sustained maintenance of memory CD8 T cell populations may serve as a foundation for protective vaccination strategies against ZIKV infections. The generation of memory CD8 T cells—including their differentiation into distinct subsets that reside in specific anatomical locations—is therefore critical for sustained immune surveillance and rapid protective responses upon re-exposure to the pathogen. The essential function of CD8 T cells in antiviral adaptive immunity is depicted in Fig. 1.

### Mechanisms of immune cell recruitment into the CNS during flavivirus infection

The CNS has traditionally been regarded as an immune-privileged site, primarily due to the protective function of the BBB, which tightly regulates the entry of immune cells and molecules [45]. Under steady-state conditions, this barrier preserves the delicate neural environment by limiting immune surveillance. However, during viral infections or systemic inflammation, BBB integrity may be compromised [46], allowing immune cell infiltration into the CNS—a response that is essential for pathogen clearance but potentially harmful if dysregulated.

Several mechanisms facilitate immune cell entry into the CNS. Inflammatory cytokines such as IL-1β, IL-6, and TNF-α disrupt the BBB by degrading tight junction proteins like claudin-5, reducing β-catenin, and damaging astrocytes, thereby increasing permeability and facilitating immune cell infiltration [47]. In parallel, another well-characterized mechanism facilitating CNS entry is the “Trojan horse”, whereby pathogens infect circulating leukocytes—such as monocytes or neutrophils—which subsequently cross the BBB and inadvertently deliver the pathogen into the CNS [48]. For instance, WNV exploits this mechanism by infecting polymorphonuclear neutrophils (PMNs) [49]. The proinflammatory chemokine osteopontin (OPN) has been shown to promote the recruitment of these infected PMNs into the brain, thereby enhancing viral neuroinvasion [50].

Several human studies have demonstrated that monocytes are the primary targets of ZIKV infection in peripheral blood [51, 52]. Upon infection, monocytes not only serve as viral reservoirs but also upregulate surface markers such as CD169 (Siglec-1), which are associated with adhesion and migration, thereby facilitating their trafficking to peripheral tissues and potentially the CNS [51]. ZIKV infection differentially reprograms monocytes, with Asian strains inducing M2-skewed IL-10–dominant responses and African strains promoting M1-type inflammation marked by CXCL10. In pregnant women, heightened viral infectivity further amplifies these immune shifts and is linked to pregnancy-related complications [52]. These findings provide a foundation for understanding the mechanism by which ZIKV spreads systemically and enters the CNS.

Emerging evidence suggests that ZIKV may similarly use a Trojan horse strategy. ZIKV-infected monocytes exhibit increased expression of adhesion molecules, enhancing their ability to adhere to and migrate across endothelial barriers. Upon entering the CNS, these monocytes can transmit the virus to resident neural cells, thereby contributing to neuropathogenesis [53]. This model is supported by findings demonstrating that ZIKV efficiently infects human primary monocytes and facilitates viral spread to cerebral organoids and brain tissue [54]. Furthermore, specific host receptors—including chemokine receptor 7 (CCR7) and the receptor for advanced glycation end products (RAGE)—have been implicated in mediating the transmigration of ZIKV-infected monocytes across the BBB [55].

Chemokines play a central role in orchestrating immune cell recruitment into the CNS during viral infections. Their upregulation within the CNS establishes chemotactic gradients that guide peripheral immune cells expressing the corresponding receptors toward infected regions [56]. A range of CNS-resident cells—including astrocytes, microglia, neurons, and brain endothelial cells—produce chemokines in response to neurotropic viral infections. For example, during WNV infection, these cells secrete chemokines such as CXCL10 and CCL5, which are crucial for the recruitment of immune effector cells to the CNS [57]. In the context of ZIKV, human brain microvascular endothelial cells (hBMECs) have been shown to secrete high levels of CCL5, a chemokine known to enhance immune cell survival and trafficking [58].

Chemokine–receptor interactions mediate the selective recruitment of distinct immune cell subsets. CXCL10, produced by infected neurons, binds to CXCR3 on activated T cells, promoting their infiltration into the CNS [59, 60]. Similarly, CCL5 interacts with CCR5 on both T cells and monocytes to facilitate their migration to inflamed brain regions [61]. Monocyte infiltration is further regulated by the CCL2–CCR2 axis. During neurotropic flavivirus infection such as Usutu virus (USUV), virus-induced CCL2 binds to CCR2 on inflammatory monocytes, promoting their recruitment into the CNS. Notably, genetic deletion of Ccr2 in mice significantly reduced monocyte accumulation in the brain and alleviated BBB disruption and neuroinflammation, highlighting the central role of this chemokine axis in viral neuropathogenesis [62]. Although CCR2 is predominantly associated with monocytes, recent evidence indicates that CD8 TRM cells in the CNS also express CCR2. In a murine model of WNV infection, CCR2 was shown to regulate TRM phenotype and function during recovery by limiting CD103 and IFN-γ expression. This modulation helped reduce neuroinflammation and preserve cognitive function, highlighting a neuroprotective role for CCR2 in CD8 T cell-mediated CNS responses [63]. Neutrophil trafficking is regulated through the CXCL1–CXCR2 pathway, as shown in Saint Louis encephalitis virus (SLEV) infection, where excessive viral replication induces CXCL1 production in the brain. This chemokine attracts CXCR2-expressing neutrophils, which may further compromise BBB integrity and amplify CNS inflammation, correlating with the early onset of neurological disease [64, 65].

While the infiltration of immune cells—particularly CD8 T cells—is essential for clearing neurotropic pathogens, this process must be tightly regulated to prevent detrimental outcomes. Antigen-specific CD8 T cells can cross the BBB in response to their cognate antigens presented by cerebral endothelial cells, enabling them to selectively eliminate infected cells while minimizing injury to healthy tissue [66]. Moreover, CD8 TRM cells have been identified within the human brain, where they contribute to rapid, localized antiviral responses [67].

**Fig. 2:**
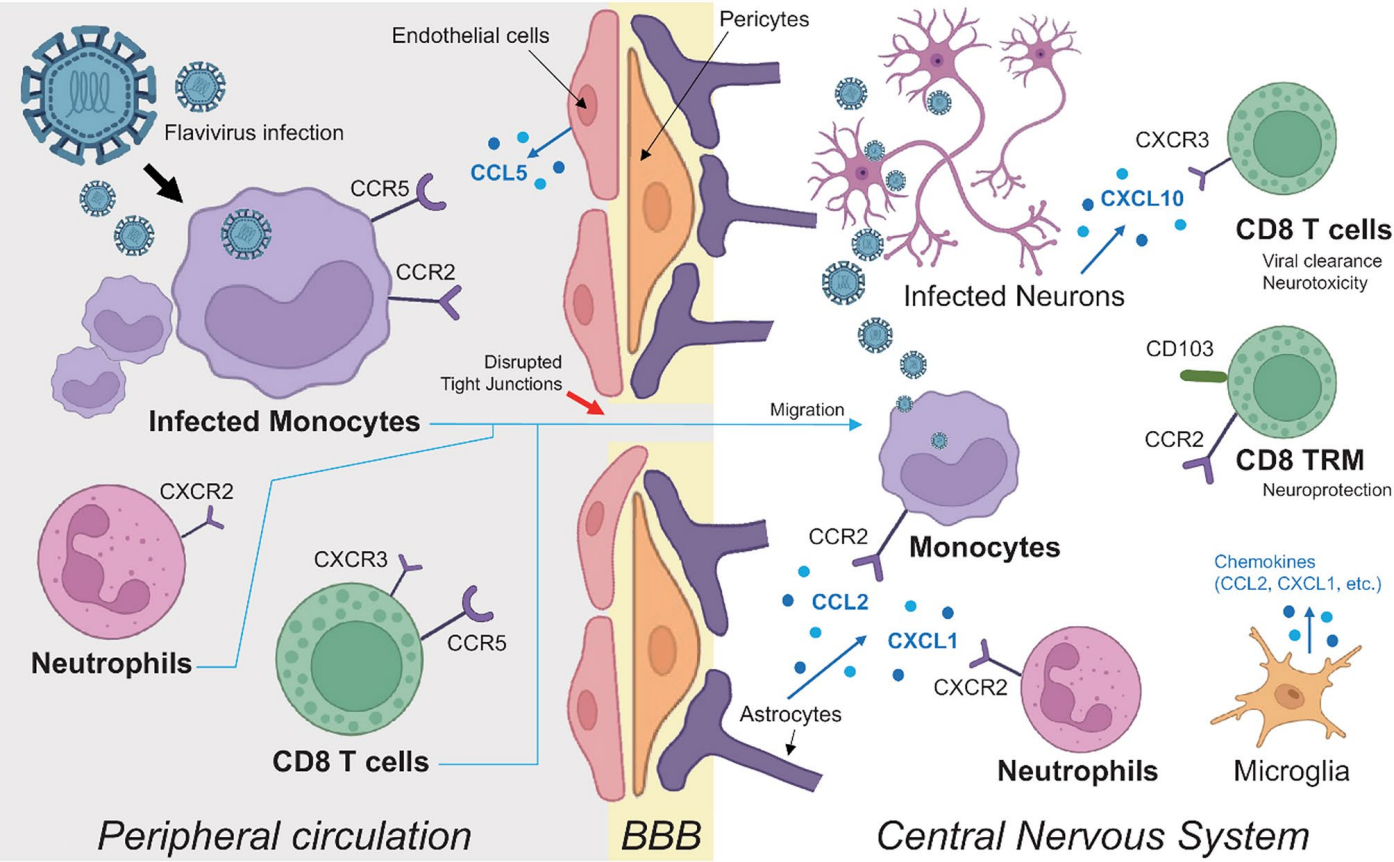
Chemokine-mediated infiltration of peripheral immune cells into the CNS during flavivirus infection. The CNS, typically an immune-privileged site, becomes accessible to peripheral immune cells during flavivirus infection through a combination of BBB disruption and chemokine-driven recruitment. Flavivirus-infected monocytes expressing CCR2 and CCR5 may cross the BBB via a “Trojan horse” mechanism, carrying viral particles into the CNS. Within the CNS, astrocytes and microglia respond to infection by producing chemokines such as CCL2 and CXCL1, which recruit monocytes and neutrophils via CCR2 and CXCR2, respectively. CD8 T cells are attracted by CXCL10 secreted by infected neurons, engaging CXCR3. A subset of CCR2-expressing CD8 T cells persists as TRM cells and exerts neuroprotective functions. Endothelial cells contribute to immune cell entry by expressing adhesion molecules and secreting CCL5, which binds to CCR5. While CD8 T cells are essential for controlling viral spread, their accumulation in the CNS may also cause collateral neuronal damage and contribute to flavivirus-associated neuropathology

Despite their protective roles, overactive or poorly regulated CD8 T cell responses within the CNS can cause severe immunopathology. These effects may include neuronal damage, reactive gliosis, and chronic neuroinflammation, potentially contributing to long-term cognitive impairment and neurodegeneration [68]. These findings underscore the importance of precise immune regulation in the CNS, where the costs of excessive inflammation can be particularly severe and often irreversible. Figure 2 summarizes how chemokines orchestrate the entry of peripheral immune cells into the CNS in the context of flavivirus infection.

### Dual roles of CD8 T cells in ZIKV infection: protection and pathology

An increasing body of research underscores the complex, dual role of CD8 T cells during ZIKV infection—not only as key effectors in controlling viral replication and promoting immune protection, but also as potential contributors to tissue damage and immunopathology [18]. This dichotomy appears especially relevant in immune-privileged sites such as the CNS and reproductive organs.

In support of their protective role, early studies using adoptive transfer models demonstrated the critical role of CD8 T cells in controlling primary ZIKV infection. Transfer of CD8 T cells from ZIKV-immune mice into T cell-deficient recipients significantly reduced viremia and tissue viral loads, confirming the protective capacity of virus-specific CD8 T cells in vivo [24, 69]. Consistently, CD8 T cell depletion in infected animals led to elevated viral replication and increased mortality, underscoring their essential role in containing viral dissemination during acute infection [24].

The antiviral efficacy of CD8 T cells is further supported by vaccine research. An alphavirus-based replicon RNA vaccine encoding the ZIKV nonstructural protein NS3 was shown to elicit robust, polyfunctional CD8 T cell responses that were both necessary and sufficient to prevent mortality in adult mice and fetal growth restriction in pregnant females [70]. These findings support the inclusion of CD8 T cell epitopes in vaccine strategies to achieve long-lasting protection, particularly in high-risk populations such as pregnant women.

In immunocompetent mouse models, ZIKV infection induces a potent CD8 T cell response, marked by the expansion of virus-specific effector and memory populations [25]. Epitope-mapping studies have identified several immunodominant peptides, and these virus-specific CD8 T cells have been shown to exert cytotoxic functions and produce key antiviral cytokines such as IFN-γ and TNF-α [24]. Notably, prior DENV infection—a related flavivirus—can elicit cross-reactive CD8 T cells that provide partial protection against subsequent ZIKV challenge, suggesting a shared immunological landscape among flaviviruses that may inform vaccine development in endemic regions [71].

Beyond their systemic antiviral functions, CD8 T cells also infiltrate the CNS during ZIKV infection. These infiltrating cells have been implicated in suppressing viral replication in the brain, and their presence is associated with reduced CNS viral burden [72]. Nevertheless, immune responses within the CNS must be tightly regulated. While beneficial for viral clearance, CD8 T cell infiltration can also amplify local inflammation and potentially contribute to neuropathology. One study by Jurado et al. reported that ZIKV infection led to breakdown of the BBB and subsequent infiltration of CD8 T cells into the CNS [17]. Although these cells limited neuronal infection, their presence was also associated with neuroinflammation and paralysis, suggesting a potential contribution to ZIKV-induced neuropathology.

Beyond the CNS, CD8 T cells are also essential for clearing ZIKV from immune-privileged tissues such as the testes, thereby reducing viral persistence and minimizing the risk of sexual transmission [73]. However, their activation has also been linked to tissue-specific pathology, including testicular inflammation and damage [73]. Several studies have drawn attention to the pathological potential of CD8 T cell responses during ZIKV infection. In murine models, CD8 T cell activity has been associated with inflammation in peripheral tissues such as the skin and eyes, presenting as auricular dermatitis and blepharitis [69]. These findings indicate that while CD8 T cells are indispensable for viral control, they may also contribute to local tissue injury.

Particularly concerning are recent studies suggesting that bystander-activated CD8 T cells—those activated independently of T cell receptor (TCR) engagement—may drive neuropathology during ZIKV infection, potentially via uncontrolled inflammatory responses. For example, Balint et al. demonstrated that neurological disease severity did not correlate with brain viral titers, but instead with the accumulation of NKG2D(+) bystander CD8 T cells. These cells contributed to neuronal damage and motor deficits, and both CD8 T cell depletion and NKG2D blockade effectively prevented ZIKV-associated paralysis [19]. These findings suggest that CD8 T cells may contribute to neuropathology through TCR-independent pathways, emphasizing the importance of tightly regulating their activity to prevent unintended tissue injury.

Collectively, these findings underscore the dual nature of CD8 T cell responses in ZIKV pathogenesis. While they are crucial for effective viral clearance and the development of protective immunity across both systemic and immune-privileged compartments, their activity must be precisely controlled to avoid immunopathological outcomes—especially within the CNS. This complexity highlights the importance of not only characterizing CD8 T cell effector functions, but also elucidating the regulatory networks that govern their activity during neurotropic viral infections. A summary of CD8 T cell functions in ZIKV infection models is presented in Table 1.

**Table 1:** Summary of CD8 T cell functions during ZIKV infection model

Model	Cell Type	Function	Effect	Reference
Mouse (T cell-deficient recipients)	CD8 T cells (from ZIKV-immune mice)	Reduced viremia and tissue viral loads	Protective	[69]
Mouse	CD8 T cells (depleted in vivo)	Increased viral replication and mortality	Protective	[24]
Mouse (vaccine study)	CD8 T cells (induced by NS3 vaccine)	Prevention of mortality and fetal growth restriction	Protective	[70]
Immunocompetent mouse	Virus-specific CD8 T cells	Cytotoxicity, cytokine production (IFN-γ, TNF-α)	Protective	[24, 25]
Mouse (prior DENV infection)	Cross-reactive CD8 T cells	Partial protection against ZIKV	Protective	[71]
Mouse (CNS infiltration)	Brain-infiltrating CD8 T cells	Suppress CNS viral replication	Protective	[72]
Mouse (testis)	CD8 T cells	Clear ZIKV, reduce sexual transmission risk	Protective/Pathological	[73]
Mouse (skin, eyes)	CD8 T cells	Auricular dermatitis, blepharitis	Pathological	[69]
Mouse (CNS)	Bystander-activated CD8 T cells	Potential neuropathology	Pathological	[19]

### Therapeutic and vaccine strategies against Zika and related flavivirus infections

In response to the ZIKV epidemic of 2015–2016, significant efforts were devoted to developing effective therapeutic interventions, though many initiatives encountered logistical and epidemiological challenges. One of the primary areas of focus was vaccine development. Among the early candidates, GLS-5700—a synthetic DNA vaccine encoding the ZIKV prM and E proteins—was developed by Inovio Pharmaceuticals and GeneOne Life Science. It entered a Phase 1 clinical trial (ZIKA-001) in 2016 to evaluate safety and immunogenicity in healthy adults. Forty participants received intradermal injections followed by electroporation using the CELLECTRA^®^ device. The vaccine was well tolerated, with no serious adverse events, and elicited binding antibodies in all participants and neutralizing antibodies in over 95% [74]. However, despite promising preclinical results, the steep decline in ZIKV cases following the epidemic rendered large-scale efficacy trials unfeasible due to insufficient ongoing transmission [75].

In parallel with vaccine development, researchers explored several antiviral agents for their efficacy against ZIKV. Sofosbuvir, a nucleotide analog inhibitor of RNA polymerase originally approved for hepatitis C virus (HCV), demonstrated the ability to inhibit replication of multiple ZIKV strains in human cell lines, including fetal neuronal stem cells. Oral administration of sofosbuvir protected mice from ZIKV-induced mortality, underscoring its therapeutic promise [76]. In a non-human primate model, sofosbuvir treatment in pregnant rhesus macaques reduced maternal viremia and significantly decreased fetal infection rates. Notably, congenital abnormalities occurred only in the offspring of untreated dams, suggesting a protective effect against congenital Zika syndrome [77].

Additional experimental approaches have shown encouraging results. Brain-penetrant antiviral peptides reduced neuroinflammation and protected neurons from ZIKV-induced injury in murine models. These peptides successfully crossed the blood–brain barrier, decreased viral loads in the brain, and preserved barrier integrity [78]. Similarly, Z2—a synthetic peptide derived from the ZIKV envelope stem region—effectively inhibited infection both in vitro and in vivo. In pregnant mice, Z2 reduced vertical transmission and supported fetal development, further underscoring its potential for preventing congenital disease [79].

Passive immunotherapy has also emerged as a promising strategy. In one study, the transfer of convalescent serum from ZIKV-infected individuals to pregnant mice not only suppressed viral replication but also prevented apoptosis and loss of neural progenitor cells in fetal mouse brains, thereby averting microcephaly [80]. In another study, high-titer human polyclonal IgG purified from convalescent donors (ZIKV-IG) was passively transferred to Ifnar1⁻/⁻ mice following lethal ZIKV challenge. This treatment significantly improved survival, reduced viral loads in peripheral and CNS tissues—including the brain—and attenuated virus-induced neuropathology [81]. These findings highlight the potential of passive immunotherapy to protect vulnerable populations, particularly in the context of congenital and neuroinvasive ZIKV disease.

Beyond direct antiviral approaches, immunomodulatory therapies have been investigated to mitigate immune-mediated pathology during ZIKV infection. Anakinra, an interleukin-1 receptor antagonist, was shown to protect against fetal neurodevelopmental deficits in infected pregnant mice by preserving placental function, improving fetal survival, and reducing microglial activation in a dose-dependent manner [82].

**Fig. 3:**
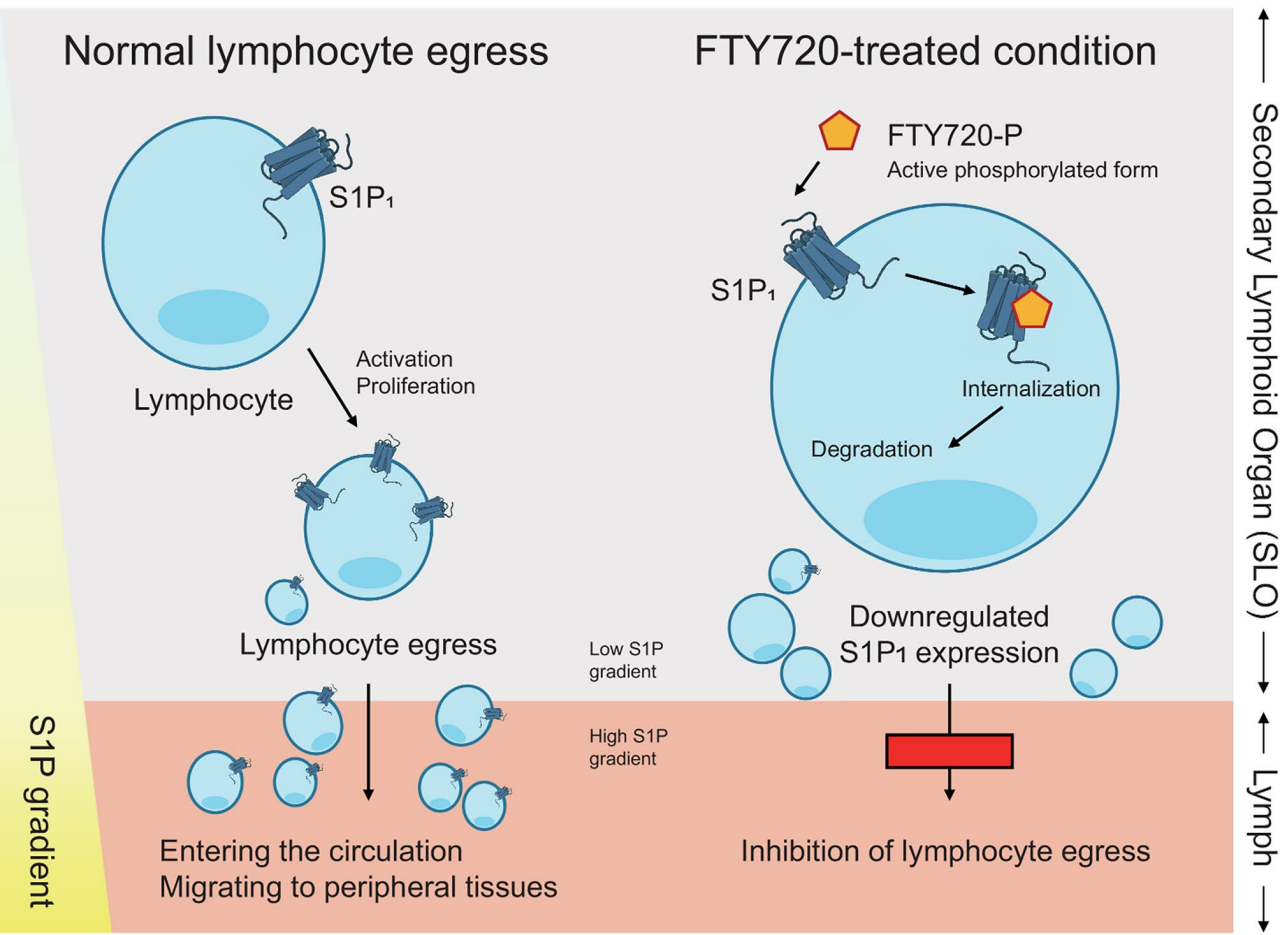
Mechanism of FTY720 (fingolimod) in regulating lymphocyte trafficking via S1P_1_ receptor modulation. Under normal physiological conditions, lymphocytes in secondary lymphoid organs (SLOs) express the S1P_1_, which senses the S1P gradient between the SLO (low S1P) and lymph/blood (high S1P). This gradient allows activated lymphocytes to exit the SLO and migrate into circulation, facilitating tissue infiltration. When FTY720 is phosphorylated into its active form (FTY720-P), it binds to S1P_1_ on lymphocytes, triggering receptor internalization and degradation. This downregulates surface S1P_1_ expression and impairs the lymphocytes’ ability to respond to the S1P gradient. As a result, lymphocyte egress is blocked, leading to their sequestration within SLOs and reduced migration to peripheral tissues, including inflamed sites such as the CNS

FTY720, a modulator of the sphingosine-1-phosphate (S1P) receptor, limits lymphocyte infiltration into the CNS by disrupting S1P1 signaling and sequestering lymphocytes within secondary lymphoid organs [83, 84] (Fig. 3). This mechanism has been demonstrated in models of neuroinflammation, including JHMV-induced encephalomyelitis and experimental autoimmune encephalomyelitis (EAE), where FTY720 treatment reduced CD4 and CD8 T cell infiltration and alleviated CNS pathology [83, 85]. In addition to T cells, FTY720 has been shown to reduce B cell accumulation and modulate resident CNS immune cells such as astrocytes and microglia, suppressing the production of proinflammatory cytokines [86, 87]. The neuroprotective potential of FTY720 has also been demonstrated in preclinical models of CNS injury and neurodegeneration. In traumatic brain injury and Alzheimer’s disease models, FTY720 preserved the BBB integrity, reduced neuroinflammation, and improved neurological outcomes [88–90]. Its clinical efficacy has been established in multiple sclerosis, where it reduces relapse rates and delays disability progression by preventing autoreactive lymphocyte egress from lymphoid tissues [91, 92].

These findings suggest that FTY720 and related S1P receptor modulators may hold promise as therapeutic agents in various CNS diseases characterized by immune cell infiltration and chronic neuroinflammation. The immunomodulatory and neuroprotective effects of FTY720 across various CNS disease models are summarized in Table 2. Although their application in flavivirus infection remains to be fully explored, the immunomodulatory and neuroprotective properties of FTY720 highlight its potential as a promising therapeutic strategy for flavivirus-associated CNS disease.

**Table 2:** Immunomodulatory effects of FTY720 across CNS disease models

Disease/Model	Targeted Cells	Effect of FTY720	Species	Reference
JHMV-induced encephalomyelitis	CD4 and CD8 T cells	Retention in lymph nodes, reduced CNS infiltration, less demyelination	Mouse	[83]
EAE (MS model)	CD4 T cells	Reduced infiltration of autoreactive T cells, disease attenuation	Mouse	[85]
EAE (MS model)	B cells	Decreased B cell infiltration into CNS	Mouse	[86]
Chronic EAE	Astrocytes and microglia	Downregulated proinflammatory cytokine/chemokine expression	Mouse	[87]
Multiple Sclerosis (clinical)	Autoreactive lymphocytes	Reduced migration, fewer relapses, delayed disability progression	Human	[91, 92]
Traumatic Brain Injury (TBI)	Endothelial and immune cells	Stabilized BBB, reduced apoptosis/inflammation, improved neurological outcomes	Mouse	[88]
Alzheimer’s Disease (3xTg-AD)	Neurons, glial cells	Reversed memory deficits, reduced tau phosphorylation and neuroinflammation	Mouse	[89]
Alzheimer’s Disease (APP/PS1)	Hippocampal neurons, glial cells	Restored synaptic function, reduced gliosis	Mouse	[90]

Insights from related flaviviruses further inform therapeutic strategies for ZIKV. In DENV infection, interferon-alpha-2a (IFN-α-2a) modestly delayed peak viremia in rhesus monkeys, though its overall antiviral efficacy was limited [93]. For WNV, although no antivirals are currently approved, promising immunotherapeutic candidates are under development. One such candidate, a potent human monoclonal antibody known as WNV-86, targets a conserved epitope on the viral envelope protein and protected mice from lethal infection when administered post-exposure [94].

Collectively, these studies underscore the complexity of developing therapies that not only suppress viral replication but also appropriately modulate host immune responses. In neurotropic flavivirus infections such as ZIKV—where immune responses can simultaneously control infection and contribute to pathology—therapeutic strategies must carefully balance CD8 T cell-mediated protection with the risk of collateral neural damage. A comparative summary of vaccine candidates and antiviral therapies for ZIKV and other flaviviruses is presented in Table 3.

**Table 3:** Therapeutic and vaccine strategies for ZIKV and related flavivirus infections

Virus	Strategy Type	Intervention	Description	Model/Trial Stage	Key Outcome	Reference
ZIKV	Vaccine	GLS-5700 DNA vaccine	Synthetic DNA vaccine encoding prM and E proteins; intradermal + electroporation	Phase 1 Clinical Trial (ZIKA-001), 2016	Safe, well-tolerated; 100% binding Ab, >95% neutralizing Ab	[74, 75]
	Antiviral	Sofosbuvir	RNA polymerase inhibitor; approved for HCV	In vitro; mouse model; rhesus macaques	Reduced replication; protected mice; reduced fetal infection and abnormalities	[76, 77]
	Antiviral Peptide	Brain-penetrating peptides	Cross BBB; reduce neuroinflammation; protect neurons	Mouse model	Reduced brain viral load and BBB injury	[78]
	Antiviral Peptide	Z2 peptide	Synthetic peptide from ZIKV envelope stem	In vitro; pregnant mouse model	Reduced vertical transmission; protected fetal development	[79]
	Passive Immunotherapy	Convalescent serum	Sera from recovered individuals containing neutralizing antibodies	Pregnant mouse model	Suppressed viral replication; prevented fetal neural damage and birth defects	[80, 81]
	Immunomodulation	Anakinra	IL-1 receptor antagonist	Pregnant mouse model	Preserved placental function; enhanced fetal survival; reduced microglial activation	[82]
DENV	Antiviral/Immunotherapy	IFN-α-2a	Broad-spectrum antiviral cytokine	Rhesus monkey model	Delayed viremia peak by 3 days; transient effect	[93]
WNV	Monoclonal Antibody	WNV-86	Targets envelope protein domain II; preferentially binds mature virions	Mouse model; post-infection administration	Protected mice from lethal challenge when given 2 days after infection	[94]

## Conclusion

ZIKV infection highlights the intricate interplay between viral pathogenesis and host immune responses, particularly within the CNS. CD8 T cells play a pivotal role in controlling viral replication and eliminating infected cells, but their activity can also trigger neuroinflammation and tissue damage if not properly regulated. This duality emphasizes the need for therapeutic strategies that carefully modulate CD8 T cell responses—maximizing antiviral efficacy while minimizing immunopathology. The immunomodulatory agent FTY720, which restricts lymphocyte infiltration into the CNS, represents a promising approach to achieving this balance.

Vaccine candidates, antiviral drugs such as sofosbuvir, and passive immunotherapies also show promise in preventing ZIKV infection and mitigating fetal neurological complications. Insights from related flavivirus infections further inform the development of immunotherapies that balance immune activation with regulation. These strategies underscore the importance of fine-tuning immune responses to prevent both viral persistence and immune-mediated damage.

Moving forward, research should focus on elucidating the regulatory networks that govern CD8 T cell function during neurotropic viral infections and on developing targeted interventions that enhance protective immunity without exacerbating CNS inflammation. Ultimately, a deeper understanding of neuroimmune dynamics will be essential for designing safe and effective treatments against ZIKV and other emerging neurotropic viruses.

## Data Availability

No datasets were generated or analysed during the current study.

## References

[1] Mackenzie JS, Gubler DJ, Petersen LR. Emerging flaviviruses: the spread and resurgence of Japanese encephalitis, West nile and dengue viruses. Nat Med. 2004;10(12 Suppl):S98–109. 10.1038/nm1144. 15577938 10.1038/nm1144

[2] Lindenbach BD, Rice CM. Molecular biology of flaviviruses. Adv Virus Res. 2003;59:23–61. 10.1016/s0065-3527(03)59002-9. 14696326 10.1016/s0065-3527(03)59002-9

[3] Mukhopadhyay S, Kuhn RJ, Rossmann MG. A structural perspective of the flavivirus life cycle. Nat Rev Microbiol. 2005;3(1):13–22. 10.1038/nrmicro1067. 15608696 10.1038/nrmicro1067

[4] Miller S, Krijnse-Locker J. Modification of intracellular membrane structures for virus replication. Nat Rev Microbiol. 2008;6(5):363–74. 10.1038/nrmicro1890. 18414501 10.1038/nrmicro1890 PMC7096853

[5] Brand C, Bisaillon M, Geiss BJ. Organization of the flavivirus Rna replicase complex. Wiley Interdiscip Rev RNA. 2017;8(6). 10.1002/wrna.1437. 10.1002/wrna.1437 PMC5675032 28815931

[6] Araujo AQ, Silva MT, Araujo AP. Zika Virus-Associated neurological disorders: A review. Brain. 2016;139(Pt 8):2122–30. 10.1093/brain/aww158. 27357348 10.1093/brain/aww158

[7] Winkelmann ER, Luo H, Wang T. West nile virus infection in the central nervous system. F1000Res. 2016;5. 10.12688/f1000research.7404.1. 10.12688/f1000research.7404.1 PMC4755400 26918172

[8] Sharma KB, Vrati S, Kalia M. Pathobiology of Japanese encephalitis virus infection. Mol Aspects Med. 2021;81:100994. 10.1016/j.mam.2021.100994. 34274157 10.1016/j.mam.2021.100994

[9] Li F, et al. Viral infection of the central nervous system and neuroinflammation precede Blood-Brain barrier disruption during Japanese encephalitis virus infection. J Virol. 2015;89(10):5602–14. 10.1128/JVI.00143-15. 25762733 10.1128/JVI.00143-15 PMC4442524

[10] Suen WW, Prow NA, Hall RA, Bielefeldt-Ohmann H. Mechanism of West nile virus neuroinvasion: A critical appraisal. Viruses. 2014;6(7):2796–825. 10.3390/v6072796. 25046180 10.3390/v6072796 PMC4113794

[11] Cle M, et al. Zika virus infection promotes local inflammation, cell adhesion molecule upregulation, and leukocyte recruitment at the Blood-Brain barrier. mBio. 2020;11(4). 10.1128/mBio.01183-20. 10.1128/mBio.01183-20 PMC7407083 32753493

[12] Sun L, Su Y, Jiao A, Wang X, Zhang B. T cells in health and disease. Signal Transduct Target Ther. 2023;8(1):235. 10.1038/s41392-023-01471-y. 37332039 10.1038/s41392-023-01471-y PMC10277291

[13] Zhang N, Bevan MJ. Cd8(+) T cells: foot soldiers of the immune system. Immunity. 2011;35(2):161–8. 10.1016/j.immuni.2011.07.010. 21867926 10.1016/j.immuni.2011.07.010 PMC3303224

[14] Garber C, et al. T cells promote Microglia-Mediated synaptic elimination and cognitive dysfunction during recovery from neuropathogenic flaviviruses. Nat Neurosci. 2019;22(8):1276–88. 10.1038/s41593-019-0427-y. 31235930 10.1038/s41593-019-0427-y PMC6822175

[15] Frieser D, et al. Tissue-Resident Cd8(+) T cells drive compartmentalized and chronic autoimmune damage against Cns neurons. Sci Transl Med. 2022;14(640):eabl6157. 10.1126/scitranslmed.abl6157. 35417189 10.1126/scitranslmed.abl6157

[16] Wang Y, Lobigs M, Lee E, Mullbacher A. Cd8 + T cells mediate recovery and immunopathology in West nile virus encephalitis. J Virol. 2003;77(24):13323–34. 10.1128/jvi.77.24.13323-13334.2003. 14645588 10.1128/JVI.77.24.13323-13334.2003 PMC296062

[17] Jurado KA, Yockey LJ, Wong PW, Lee S, Huttner AJ, Iwasaki A. Antiviral Cd8 T cells induce Zika-Virus-Associated paralysis in mice. Nat Microbiol. 2018;3(2):141–7. 10.1038/s41564-017-0060-z. 29158604 10.1038/s41564-017-0060-z PMC5780207

[18] Pardy RD, Richer MJ. Protective to a T: the role of T cells during Zika virus infection. Cells. 2019;8(8). 10.3390/cells8080820. 10.3390/cells8080820 PMC6721718 31382545

[19] Balint E, et al. Bystander activated Cd8(+) T cells mediate neuropathology during viral infection via Antigen-Independent cytotoxicity. Nat Commun. 2024;15(1):896. 10.1038/s41467-023-44667-0. 38316762 10.1038/s41467-023-44667-0 PMC10844499

[20] Maximova OA, Pletnev AG. Flaviviruses and the central nervous system: revisiting neuropathological concepts. Annu Rev Virol. 2018;5(1):255–72. 10.1146/annurev-virology-092917-043439. 30265628 10.1146/annurev-virology-092917-043439

[21] Cody SG, Adam A, Siniavin A, Kang SS, Wang T. Flaviviruses-Induced Neurol Sequelae Pathogens. 2024;14(1). 10.3390/pathogens14010022. 10.3390/pathogens14010022 PMC11768111 39860983

[22] Mlakar J, et al. Zika virus associated with microcephaly. N Engl J Med. 2016;374(10):951–8. 10.1056/NEJMoa1600651. 26862926 10.1056/NEJMoa1600651

[23] Franca GV, et al. Congenital Zika virus syndrome in brazil: A case series of the first 1501 livebirths with complete investigation. Lancet. 2016;388(10047):891–7. 10.1016/S0140-6736(16)30902-3. 27372398 10.1016/S0140-6736(16)30902-3

[24] Elong Ngono A, et al. Mapping and role of the Cd8(+) T cell response during primary Zika virus infection in mice. Cell Host Microbe. 2017;21(1):35–46. 10.1016/j.chom.2016.12.010. 28081442 10.1016/j.chom.2016.12.010 PMC5234855

[25] Huang H, et al. Cd8(+) T cell immune response in immunocompetent mice during Zika virus infection. J Virol. 2017;91(22). 10.1128/JVI.00900-17. 10.1128/JVI.00900-17 PMC5660488 28835502

[26] Pardy RD, Rajah MM, Condotta SA, Taylor NG, Sagan SM, Richer MJ. Analysis of the T cell response to Zika virus and identification of a novel Cd8 + T cell epitope in immunocompetent mice. PLoS Pathog. 2017;13(2):e1006184. 10.1371/journal.ppat.1006184. 28231312 10.1371/journal.ppat.1006184 PMC5322871

[27] van Lier RA, ten Berge IJ, Gamadia LE. Human Cd8(+) T-Cell differentiation in response to viruses. Nat Rev Immunol. 2003;3(12):931–9. 10.1038/nri1254. 14647475 10.1038/nri1254

[28] Belz G, Mount A, Masson F. Dendritic cells in viral infections. Handb Exp Pharmacol. 2009;188:51–77. 10.1007/978-3-540-71029-5_3. 10.1007/978-3-540-71029-5_3 19031021

[29] Hartmann BM, Marjanovic N, Nudelman G, Moran TM, Sealfon SC. Combinatorial cytokine code generates Anti-Viral state in dendritic cells. Front Immunol. 2014;5:73. 10.3389/fimmu.2014.00073. 24616721 10.3389/fimmu.2014.00073 PMC3935347

[30] Voskoboinik I, Whisstock JC, Trapani JA. Perforin and granzymes: function, dysfunction and human pathology. Nat Rev Immunol. 2015;15(6):388–400. 10.1038/nri3839. 25998963 10.1038/nri3839

[31] Lopez JA, et al. Perforin forms transient pores on the target cell plasma membrane to facilitate rapid access of granzymes during killer cell attack. Blood. 2013;121(14):2659–68. 10.1182/blood-2012-07-446146. 23377437 10.1182/blood-2012-07-446146

[32] Lieberman J. The Abcs of Granule-Mediated cytotoxicity: new weapons in the arsenal. Nat Rev Immunol. 2003;3(5):361–70. 10.1038/nri1083. 12766758 10.1038/nri1083

[33] Lowin B, Hahne M, Mattmann C, Tschopp J. Cytolytic T-Cell cytotoxicity is mediated through Perforin and Fas lytic pathways. Nature. 1994;370(6491):650–2. 10.1038/370650a0. 7520535 10.1038/370650a0

[34] Brincks EL, Katewa A, Kucaba TA, Griffith TS, Legge KL. Cd8 T cells utilize trail to control influenza virus infection. J Immunol. 2008;181(7):4918–25. 10.4049/jimmunol.181.7.4918. 18802095 10.4049/jimmunol.181.7.4918 PMC2610351

[35] Harty JT, Tvinnereim AR, White DW. Cd8 + T cell effector mechanisms in resistance to infection. Annu Rev Immunol. 2000;18:275–308. 10.1146/annurev.immunol.18.1.275. 10837060 10.1146/annurev.immunol.18.1.275

[36] Abbas AK. The surprising story of Il-2: from experimental models to clinical application. Am J Pathol. 2020;190(9):1776–81. 10.1016/j.ajpath.2020.05.007. 32828360 10.1016/j.ajpath.2020.05.007

[37] Kahan SM, et al. Intrinsic Il-2 production by effector Cd8 T cells affects Il-2 signaling and promotes fate decisions, stemness, and protection. Sci Immunol. 2022;7(68):eabl6322. 10.1126/sciimmunol.abl6322. 35148200 10.1126/sciimmunol.abl6322 PMC8923238

[38] Malek TR. The biology of Interleukin-2. Annu Rev Immunol. 2008. 10.1146/annurev.immunol.26.021607.090357. 26(453– 79. 18062768 10.1146/annurev.immunol.26.021607.090357

[39] Maurer M, von Stebut E. Macrophage inflammatory Protein-1. Int J Biochem Cell Biol. 2004;36(10):1882–6. 10.1016/j.biocel.2003.10.019. 15203102 10.1016/j.biocel.2003.10.019

[40] Schall TJ, Bacon K, Toy KJ, Goeddel DV. Selective attraction of monocytes and T lymphocytes of the memory phenotype by cytokine Rantes. Nature. 1990;347(6294):669–71. 10.1038/347669a0. 1699135 10.1038/347669a0

[41] Hamilton JA. Colony-Stimulating factors in inflammation and autoimmunity. Nat Rev Immunol. 2008;8(7):533–44. 10.1038/nri2356. 18551128 10.1038/nri2356

[42] Cox MA, Kahan SM, Zajac AJ. Anti-Viral Cd8 T cells and the cytokines that they love. Virology. 2013;435(1):157–69. 10.1016/j.virol.2012.09.012. 23217625 10.1016/j.virol.2012.09.012 PMC3580945

[43] Wherry EJ, Ahmed R. Memory Cd8 T-Cell differentiation during viral infection. J Virol. 2004;78(11):5535–45. 10.1128/JVI.78.11.5535-5545.2004. 15140950 10.1128/JVI.78.11.5535-5545.2004 PMC415833

[44] Ahlers JD, Belyakov IM. Memories that last forever: strategies for optimizing vaccine T-Cell memory. Blood. 2010;115(9):1678–89. 10.1182/blood-2009-06-227546. 19903895 10.1182/blood-2009-06-227546 PMC2920202

[45] Muldoon LL, et al. Immunologic privilege in the central nervous system and the Blood-Brain barrier. J Cereb Blood Flow Metab. 2013;33(1):13–21. 10.1038/jcbfm.2012.153. 23072749 10.1038/jcbfm.2012.153 PMC3597357

[46] Galea I. The Blood-Brain barrier in systemic infection and inflammation. Cell Mol Immunol. 2021;18(11):2489–501. 10.1038/s41423-021-00757-x. 34594000 10.1038/s41423-021-00757-x PMC8481764

[47] Gryka-Marton M, Grabowska AD, Szukiewicz D. Breaking the barrier: the role of Proinflammatory cytokines in Bbb dysfunction. Int J Mol Sci. 2025;26(8). 10.3390/ijms26083532. 10.3390/ijms26083532 PMC12026921 40331982

[48] Kim KS. Mechanisms of microbial traversal of the Blood-Brain barrier. Nat Rev Microbiol. 2008;6(8):625–34. 10.1038/nrmicro1952. 18604221 10.1038/nrmicro1952 PMC5206914

[49] Bai F, et al. A Paradoxical role for neutrophils in the pathogenesis of West nile virus. J Infect Dis. 2010;202(12):1804–12. 10.1086/657416. 21050124 10.1086/657416 PMC3053000

[50] Paul AM, et al. Osteopontin facilitates West nile virus neuroinvasion via neutrophil Trojan horse transport. Sci Rep. 2017;7(1):4722. 10.1038/s41598-017-04839-7. 28680095 10.1038/s41598-017-04839-7 PMC5498593

[51] Michlmayr D, et al. Comprehensive Immunoprofiling of pediatric Zika reveals key role for monocytes in the acute phase and no effect of prior dengue virus infection. Cell Rep. 2020;31(4):107569. 10.1016/j.celrep.2020.107569. 32348760 10.1016/j.celrep.2020.107569 PMC7308490

[52] Foo SS, et al. Asian Zika virus strains target Cd14(+) blood monocytes and induce M2-Skewed immunosuppression during pregnancy. Nat Microbiol. 2017;2(11):1558–70. 10.1038/s41564-017-0016-3. 28827581 10.1038/s41564-017-0016-3 PMC5678934

[53] Ayala-Nunez NV, et al. Zika virus enhances monocyte adhesion and transmigration favoring viral dissemination to neural cells. Nat Commun. 2019;10(1):4430. 10.1038/s41467-019-12408-x. 31562326 10.1038/s41467-019-12408-x PMC6764950

[54] Tan LY, Komarasamy TV, James W, Balasubramaniam V. Host molecules regulating neural invasion of Zika virus and drug repurposing strategy. Front Microbiol. 2022;13:743147. 10.3389/fmicb.2022.743147. 35308394 10.3389/fmicb.2022.743147 PMC8931420

[55] de Carvalho GC, Borget MY, Bernier S, Garneau D, da Silva Duarte AJ, Dumais N. Rage and Ccr7 mediate the transmigration of Zika-Infected monocytes through the Blood-Brain barrier. Immunobiology. 2019;224(6):792–803. 10.1016/j.imbio.2019.08.007. 31493920 10.1016/j.imbio.2019.08.007

[56] Griffith JW, Sokol CL, Luster AD. Chemokines and chemokine receptors: positioning cells for host defense and immunity. Annu Rev Immunol. 2014;32:659–702. 10.1146/annurev-immunol-032713-120145. 24655300 10.1146/annurev-immunol-032713-120145

[57] Hosking MP, Lane TE. The role of chemokines during viral infection of the Cns. PLoS Pathog. 2010;6(7):e1000937. 10.1371/journal.ppat.1000937. 20686655 10.1371/journal.ppat.1000937 PMC2912390

[58] Mladinich MC, Schwedes J, Mackow ER. Zika virus persistently infects and is basolaterally released from primary human brain microvascular endothelial cells. mBio. 2017;8(4). 10.1128/mBio.00952-17. 10.1128/mBio.00952-17 PMC5513708 28698279

[59] Klein RS, et al. Neuronal Cxcl10 directs Cd8 + T-Cell recruitment and control of West nile virus encephalitis. J Virol. 2005;79(17):11457–66. 10.1128/JVI.79.17.11457-11466.2005. 16103196 10.1128/JVI.79.17.11457-11466.2005 PMC1193600

[60] Zhang B, Chan YK, Lu B, Diamond MS, Klein RS. Cxcr3 mediates Region-Specific antiviral T cell trafficking within the central nervous system during West nile virus encephalitis. J Immunol. 2008;180(4):2641–9. 10.4049/jimmunol.180.4.2641. 18250476 10.4049/jimmunol.180.4.2641

[61] Glass WG, Lim JK, Cholera R, Pletnev AG, Gao JL, Murphy PM. Chemokine receptor Ccr5 promotes leukocyte trafficking to the brain and survival in West nile virus infection. J Exp Med. 2005;202(8):1087–98. 10.1084/jem.20042530. 16230476 10.1084/jem.20042530 PMC2213214

[62] Slowikowski E, et al. A central role for Ccr2 in monocyte recruitment and Blood-Brain barrier disruption during Usutu virus encephalitis. J Neuroinflammation. 2025;22(1):107. 10.1186/s12974-025-03435-1. 40241134 10.1186/s12974-025-03435-1 PMC12004732

[63] Ai S, Arutyunov A, Liu J, Hill JD, Jiang X, Klein RS. Ccr2 restricts Ifn-Gamma production by hippocampal Cd8 Trm cells that impair learning and memory during recovery from Wnv encephalitis. J Neuroinflammation. 2024;21(1):330. 10.1186/s12974-024-03309-y. 39725999 10.1186/s12974-024-03309-y PMC11673327

[64] Marques RE, et al. Development of a model of saint Louis encephalitis infection and disease in mice. J Neuroinflammation. 2017;14(1):61. 10.1186/s12974-017-0837-2. 28330482 10.1186/s12974-017-0837-2 PMC5361699

[65] Rocha RF, et al. Type I interferons are essential while type Ii interferon is dispensable for protection against st. Louis encephalitis virus infection in the mouse brain. Virulence. 2021;12(1):244–59. 10.1080/21505594.2020.1869392. 33410731 10.1080/21505594.2020.1869392 PMC7808420

[66] Galea I, Bernardes-Silva M, Forse PA, van Rooijen N, Liblau RS, Perry VH. An Antigen-Specific pathway for Cd8 T cells across the Blood-Brain barrier. J Exp Med. 2007;204(9):2023–30. 10.1084/jem.20070064. 17682068 10.1084/jem.20070064 PMC2118703

[67] Smolders J, et al. Tissue-Resident memory T cells populate the human brain. Nat Commun. 2018;9(1):4593. 10.1038/s41467-018-07053-9. 30389931 10.1038/s41467-018-07053-9 PMC6214977

[68] Vincenti I, et al. Tissue-Resident memory Cd8(+) T cells cooperate with Cd4(+) T cells to drive compartmentalized immunopathology in the Cns. Sci Transl Med. 2022;14(640):eabl6058. 10.1126/scitranslmed.abl6058. 35417190 10.1126/scitranslmed.abl6058

[69] Lee CY, et al. Cd8 + T cells trigger auricular dermatitis and blepharitis in mice after Zika virus infection in the absence of Cd4 + T cells. J Invest Dermatol. 2023;143(6):1031–e418. 10.1016/j.jid.2022.11.020. 36566875 10.1016/j.jid.2022.11.020

[70] Elong Ngono A, et al. Cd8(+) T cells mediate protection against Zika virus induced by an Ns3-Based vaccine. Sci Adv. 2020;6(45). 10.1126/sciadv.abb2154. 10.1126/sciadv.abb2154 PMC7673678 33148638

[71] Wen J, et al. Dengue virus-Reactive Cd8(+) T cells mediate Cross-Protection against subsequent Zika virus challenge. Nat Commun. 2017;8(1):1459. 10.1038/s41467-017-01669-z. 29129917 10.1038/s41467-017-01669-z PMC5682281

[72] Nazerai L et al. Effector Cd8 T Cell-Dependent Zika Virus Control in the Cns: A Matter of Time and Numbers. Front Immunol. 2020;11(1977. 10.3389/fimmu.2020.01977 10.3389/fimmu.2020.01977 PMC7461798 32973802

[73] Campos RK, et al. Cd8(+) T cells promote Zikv clearance and mitigate testicular damage in mice. Npj Viruses. 2024;2(1):20. 10.1038/s44298-024-00033-5. 40295722 10.1038/s44298-024-00033-5 PMC11721072

[74] Tebas P, et al. Safety and immunogenicity of an Anti-Zika virus DNA vaccine. N Engl J Med. 2021;385(12):e35. 10.1056/NEJMoa1708120. 34525286 10.1056/NEJMoa1708120 PMC6824915

[75] Barrett ADT. Current status of Zika vaccine development: Zika vaccines advance into clinical evaluation. NPJ Vaccines. 2018;3:24. 10.1038/s41541-018-0061-9. 29900012 10.1038/s41541-018-0061-9 PMC5995964

[76] Bullard-Feibelman KM, et al. The Fda-Approved drug Sofosbuvir inhibits Zika virus infection. Antiviral Res. 2017. 10.1016/j.antiviral.2016.11.023. 137(134– 40. 27902933 10.1016/j.antiviral.2016.11.023 PMC5182171

[77] Gardinali NR, et al. Sofosbuvir shows a protective effect against vertical transmission of Zika virus and the associated congenital syndrome in Rhesus monkeys. Antiviral Res. 2020;182:104859. 10.1016/j.antiviral.2020.104859. 32649965 10.1016/j.antiviral.2020.104859

[78] Jackman JA, et al. Therapeutic treatment of Zika virus infection using a Brain-Penetrating antiviral peptide. Nat Mater. 2018;17(11):971–7. 10.1038/s41563-018-0194-2. 30349030 10.1038/s41563-018-0194-2

[79] Yu Y, et al. A Peptide-Based viral inactivator inhibits Zika virus infection in pregnant mice and fetuses. Nat Commun. 2017;8:15672. 10.1038/ncomms15672. 28742068 10.1038/ncomms15672 PMC5537589

[80] Wang S, et al. Transfer of convalescent serum to pregnant mice prevents Zika virus infection and microcephaly in offspring. Cell Res. 2017;27(1):158–60. 10.1038/cr.2016.144. 27922617 10.1038/cr.2016.144 PMC5223229

[81] Branche E, et al. Human polyclonal antibodies prevent lethal Zika virus infection in mice. Sci Rep. 2019;9(1):9857. 10.1038/s41598-019-46291-9. 31285451 10.1038/s41598-019-46291-9 PMC6614477

[82] Lei J, et al. Il-1 receptor antagonist therapy mitigates placental dysfunction and perinatal injury following Zika virus infection. JCI Insight. 2019;4(7). 10.1172/jci.insight.122678. 10.1172/jci.insight.122678 PMC6483652 30944243

[83] Blanc CA, Rosen H, Lane TE. Fty720 (Fingolimod) modulates the severity of Viral-Induced encephalomyelitis and demyelination. J Neuroinflammation. 2014;11:138. 10.1186/s12974-014-0138-y. 25138356 10.1186/s12974-014-0138-y PMC4148542

[84] Cyster JG, Schwab SR. Sphingosine-1-Phosphate and lymphocyte egress from lymphoid organs. Annu Rev Immunol. 2012;30:69–94. 10.1146/annurev-immunol-020711-075011. 22149932 10.1146/annurev-immunol-020711-075011

[85] Chiba K, et al. Fingolimod (Fty720), sphingosine 1-Phosphate receptor modulator, shows superior efficacy as compared with Interferon-Beta in mouse experimental autoimmune encephalomyelitis. Int Immunopharmacol. 2011;11(3):366–72. 10.1016/j.intimp.2010.10.005. 20955831 10.1016/j.intimp.2010.10.005

[86] Bail K, et al. Differential effects of Fty720 on the B cell compartment in a mouse model of multiple sclerosis. J Neuroinflammation. 2017;14(1):148. 10.1186/s12974-017-0924-4. 28738885 10.1186/s12974-017-0924-4 PMC5525315

[87] Rothhammer V, et al. Sphingosine 1-Phosphate receptor modulation suppresses pathogenic astrocyte activation and chronic progressive Cns inflammation. Proc Natl Acad Sci U S A. 2017;114(8):2012–7. 10.1073/pnas.1615413114. 28167760 10.1073/pnas.1615413114 PMC5338419

[88] Cheng H, et al. Fty720 reduces endothelial cell apoptosis and remodels neurovascular unit after experimental traumatic brain injury. Int J Med Sci. 2021;18(2):304–13. 10.7150/ijms.49066. 33390799 10.7150/ijms.49066 PMC7757143

[89] Fagan SG, Bechet S, Dev KK. Fingolimod rescues memory and improves pathological hallmarks in the 3xtg-Ad model of alzheimer’s disease. Mol Neurobiol. 2022;59(3):1882–95. 10.1007/s12035-021-02613-5. 35031916 10.1007/s12035-021-02613-5 PMC8882098

[90] Kartalou GI, et al. Anti-Inflammatory treatment with Fty720 starting after onset of symptoms reverses synaptic deficits in an ad mouse model. Int J Mol Sci. 2020;21(23). 10.3390/ijms21238957. 10.3390/ijms21238957 PMC7734581 33255764

[91] Lublin F, et al. Oral Fingolimod in primary progressive multiple sclerosis (Informs): A phase 3, randomised, Double-Blind, Placebo-Controlled trial. Lancet. 2016;387(10023):1075–84. 10.1016/S0140-6736(15)01314-8. 26827074 10.1016/S0140-6736(15)01314-8

[92] Cohen JA, et al. Oral Fingolimod or intramuscular interferon for relapsing multiple sclerosis. N Engl J Med. 2010;362(5):402–15. 10.1056/NEJMoa0907839. 20089954 10.1056/NEJMoa0907839

[93] Ajariyakhajorn C, et al. Randomized, Placebo-Controlled trial of nonpegylated and pegylated forms of Recombinant human alpha interferon 2a for suppression of dengue virus viremia in Rhesus monkeys. Antimicrob Agents Chemother. 2005;49(11):4508–14. 10.1128/AAC.49.11.4508-4514.2005. 16251289 10.1128/AAC.49.11.4508-4514.2005 PMC1280153

[94] Goo L, et al. A protective human monoclonal antibody targeting the West nile virus E protein preferentially recognizes mature virions. Nat Microbiol. 2019;4(1):71–7. 10.1038/s41564-018-0283-7. 30455471 10.1038/s41564-018-0283-7 PMC6435290

